# In situ chemoimmunotherapy hydrogel elicits immunogenic cell death and evokes efficient antitumor immune response

**DOI:** 10.1186/s12967-024-05102-0

**Published:** 2024-04-09

**Authors:** Qin Liu, Rui Xu, Jingwen Shen, Yaping Tao, Jingyi Shao, Yaohua Ke, Baorui Liu

**Affiliations:** The Comprehensive Cancer Centre, Nanjing Drum Tower Hospital, Affiliated Hospital of Medical School, Nanjing University, Nanjing, China

**Keywords:** Chemoimmunotherapy, Hydrogel, Nab-PTX, TLR7 agonist, In situ vaccine

## Abstract

**Background:**

Chemoimmunotherapy has shown promising advantages of eliciting immunogenic cell death and activating anti-tumor immune responses. However, the systemic toxicity of chemotherapy and tumor immunosuppressive microenvironment limit the clinical application.

**Methods:**

Here, an injectable sodium alginate hydrogel (ALG) loaded with nanoparticle albumin-bound-paclitaxel (Nab-PTX) and an immunostimulating agent R837 was developed for local administration. Two murine hepatocellular carcinoma and breast cancer models were established. The tumor-bearing mice received the peritumoral injection of R837/Nab-PTX/ALG once a week for two weeks. The antitumor efficacy, the immune response, and the tumor microenvironment were investigated.

**Results:**

This chemoimmunotherapy hydrogel with sustained-release character was proven to have significant effects on killing tumor cells and inhibiting tumor growth. Peritumoral injection of our hydrogel caused little harm to normal organs and triggered a potent antitumor immune response against both hepatocellular carcinoma and breast cancer. In the tumor microenvironment, enhanced immunogenic cell death induced by the combination of Nab-PTX and R837 resulted in 3.30-fold infiltration of effector memory T cells and upregulation of 20 biological processes related to immune responses.

**Conclusions:**

Our strategy provides a novel insight into the combination of chemotherapy and immunotherapy and has the potential for clinical translation.

## Introduction

Chemotherapy, as a conventional treatment modality for advanced cancer, has recently been reported to be closely related to the immune system. Accumulating evidence proved that chemotherapeutic drugs have dual effects on antitumor immune responses [1]. Some of them targeting fast-dividing cells, such as gemcitabine and 5-fluorouracil, can activate immune regulatory cells, resulting in immunosuppression of the body and limiting the antitumor efficacy [2]. On the other hand, some of them can induce immunogenic cell death (ICD), inducing exposing calreticulin (CRT), releasing damage-related molecular pattern (DAMP) tumor antigens such as high-mobility group box 1 protein (HMGB1), and enhancing tumor immunogenicity [3–5]. For instance, paclitaxel, an antineoplastic agent, has been demonstrated to be capable of activating dendritic cells (DCs), altering the M2-like signature of tumor-associated macrophages toward an M1-like profile and inhibiting regulatory T cells, so it is proved to be a candidate inducer of ICD [6–9]. Furthermore, nanoparticle albumin-bound-paclitaxel (Nab-PTX) has shown better performance in some clinical trials compared with PTX, due to that Nab-PTX does not need to be pretreated with steroids to prevent allergic reaction and can be enriched at tumor sites [10].

However, chemotherapy alone may aggravate the immunosuppression of tumor microenvironment and difficult to exert a real curative effect [11]. Immunotherapy can solve this problem to some extent [12]. Immunostimulating agents with a strong ability to activate adaptive immunity, if efficiently delivered into the tumor, may effectively improve the microenvironment. In recent studies by us and other groups, Toll-like receptor 7 (TLR7) agonist imiquimod (R837) and TLR9 agonist CpG oligodeoxynucleotides (CpG-ODNs) could help make the tumor into an “in situ vaccine” after intratumoral administration with them at low doses, greatly enhancing antitumor immune responses and inhibiting both treated and distant tumors lesions [13, 14]. When Nab-PTX induces the apoptosis of tumor cells to release immunogenic antigens, the Toll-like receptor (TLR) agonist as an appealing agent of vaccine design, can help promote DC maturation, migration, antigen presentation and induce tumor-specific T cells [15, 16]. As a result, R837 can be considered as an effective combinatorial partner of chemotherapy and be introduced to our combination strategy design.

Systemic administration is the most common way for chemotherapeutic drugs to eliminate tumor cells throughout the body in the clinic but usually causes notable toxicity in normal tissues. In the setting of combining chemotherapy and immunotherapy (chemoimmunotherapy), local administration may also activate systemic antitumor immune responses to achieve similar or better efficacy and minimize systematic toxicity [17]. Compared with the traditional intravenous administration, intratumoral and peritumoral administration greatly increased the drug concentration in tumors and tumor-draining lymph nodes, which are the main source of tumor-infiltrating lymphocytes, and stimulated more potent T cell responses [18]. With the help of hydrogels, which have sustained-release property, the frequency of local administration can be effectively reduced and the safety can be further improved [19].

**Fig. 1:**
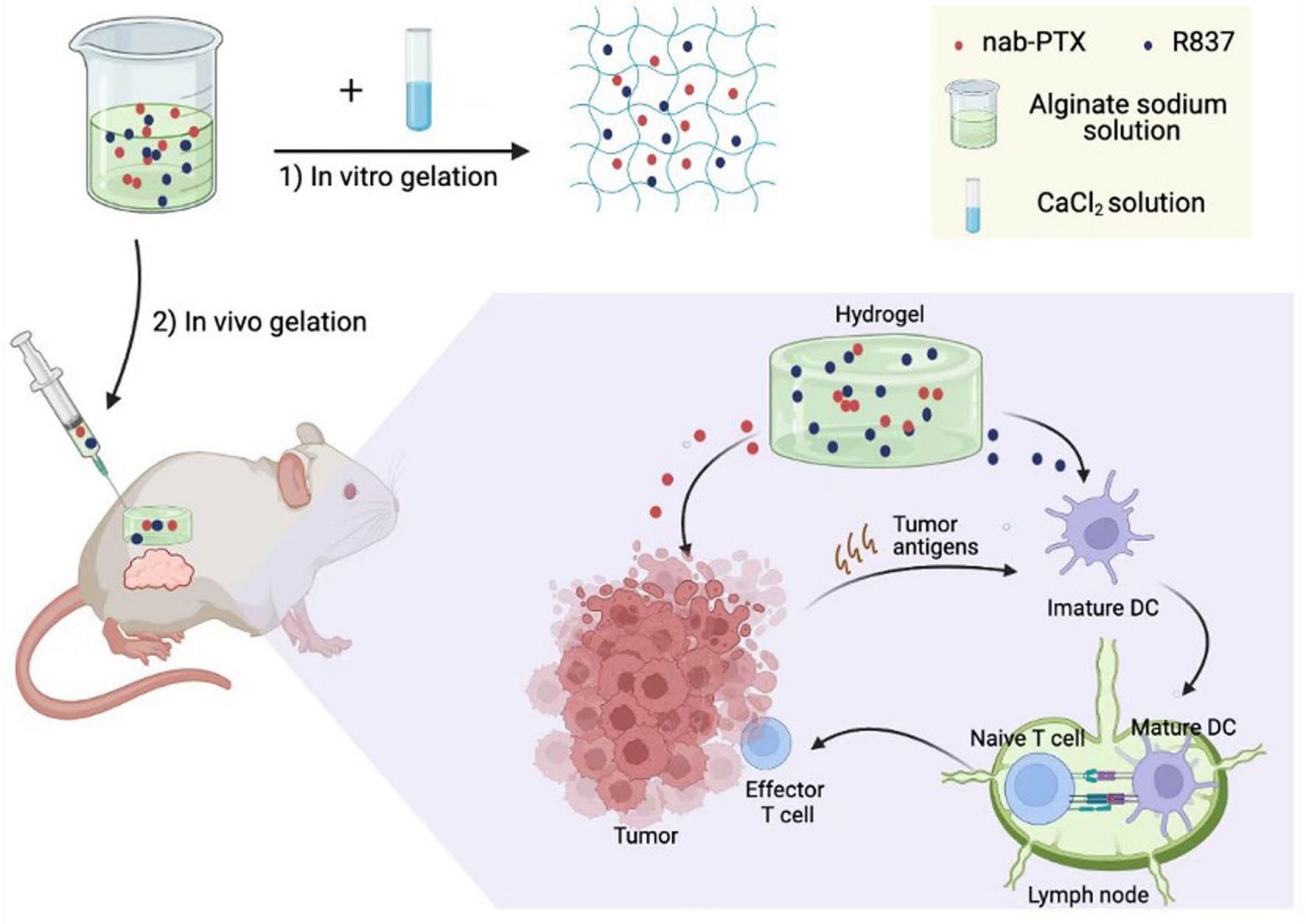
Schematic diagram of a chemoimmunotherapy hydrogel to play an anti-tumor role by reprogramming the tumor microenvironment. 1) In vitro, CaCl_2_ (1.8 × 10^–3^ mol L^−1^) is added to sodium alginate solution (ALG, 20 mg mL^−1^) mixed with nanoparticle albumin-bound-paclitaxel (Nab-PTX, 1 mg mL^−1^) and an immunostimulating agent R837 (0.5 mg mL^–1^), to form our chemoimmunotherapy hydrogel at room temperature. 2) ALG mixed with Nab-PTX and R837, will be cross-linked with the cations present in vivo to form hydrogel near the tumor. Then, some dying tumor cells release antigens due to immunogenic cell death induced by Nab-PTX. Immature DCs are activated by immunogenic tumor antigens accompanied by R837, and migrate to lymph nodes to induce sufficient effector memory T cells (T_EM_), eventually increasing the proportion of T_EM_ in tumor microenvironment

In this work, we prepared a novel chemoimmunotherapy hydrogel to make the tumor-suppressive immune microenvironment into an immunostimulatory one (Fig. 1). Sodium alginate (ALG) solution mixed with Nab-PTX and R837, can rapidly form a hydrogel near the tumor after peritumoral injection, which slowly sustained the release of Nab-PTX and R837. Then, some tumor cells expose antigens due to ICD induced by Nab-PTX. Immature DCs are then activated by immunogenic tumor antigens companied with an immunostimulating agent R837, and migrate to lymph nodes to induce sufficient effector memory T cells (T_EM_), eventually increasing the proportion of T_EM_ in tumor microenvironment. Furthermore, the slow-release effect of peritumoral hydrogel can greatly reduce the potential systemic toxicity of Nab-PTX. The combination of chemotherapy and immunotherapy exerted potent antitumor efficacy and prolonged animal survival in both murine hepatocellular carcinoma and breast cancer models. Overall, our findings indicate the clinical translation potential of the chemoimmunotherapy hydrogel.

## Materials and methods

### Materials, cells and animals

Sodium ALG was purchased from Sangon Biotech (Shanghai) Co., Ltd. Calcium chloride (CaCl_2_) was purchased from Sigma-Aldrich and R837 was purchased from BIOFOUNT (Beijing, China). Nab-PTX was provided by Shijiazhuang Pharmaceutical Group Ouyi Pharmaceutical Co., Ltd (Shijiazhuang, China). 4T1 breast cancer cells and H22 hepatocellular carcinoma cells were obtained from the Cell Bank of Shanghai Institute of Biochemistry and Cell Biology, and cultured with RPMI 1640 medium containing 10% FBS and 1% penicillin/streptomycin (37 °C, 5% CO_2_). 293 T cells were obtained from the Cell Bank of Shanghai Institute of Biochemistry and Cell Biology, and cultured with Dulbecco’s modified Eagle’s medium (DMEM) medium containing 10% FBS and 1% penicillin/streptomycin (37 °C, 5% CO_2_). Balb/c and ICR female mice aged 5–6 weeks, C57BL/6 male mice aged 8–10 weeks were purchased from Shanghai Sippr-BK Laboratory Animal Co. Ltd. (Shanghai, China) and kept in the specific pathogen-free (SPF) Laboratory Animal Center of Affiliated Nanjing Drum Tower Hospital of Nanjing University Medical School. All animal experimental protocols were approved by the Laboratory Animal Care and Use Committee of the Affiliated Nanjing Drum Tower Hospital of Nanjing University Medical School (2020AE01029).

### Preparation and characterization of R837/Nab-PTX/ALG

In vitro, CaCl_2_ (1.8 × 10^–3^ mol L^−1^) was added to sodium ALG solution (20 mg mL^−1^) mixed with Nab-PTX (1 mg mL^−1^) and an immune adjuvant R837 (0.5 mg mL^−1^), to form R837/Nab-PTX/ALG at room temperature with 10 s. In vivo, sodium ALG solution (20 mg mL^−1^) mixed with Nab-PTX (1 mg mL^−1^) and R837 (0.5 mg mL^−1^) formed R837/Nab-PTX/ALG rapidly after peritumoral injection. Scanning electron microscopy (HITACHI) was used to observe the morphology of ALG hydrogels at an accelerating voltage of 15 kV. The hydrogel was lyophilized and coated with a thin layer of gold prior to observation.

### In vivo drug release from ALG hydrogel

NIR imaging was used to detect the release of Cy5 dye (0.1 mg mL^−1^, simulating drugs) in vivo when loaded in ALG hydrogel. The tumor-bearing ICR mice were anesthetized and scanned using CRi Maestro In Vivo Imaging System (Cambridge Research & Instrumentation, Massachusetts, USA) at indicated time points (1 h, 4 h, 12 h, 48 h, 72 h, 120 h) after peritumoral injection with Cy5 (10 µg Cy5 in 100 µL normal saline) or Cy5/ALG (10 µg Cy5 in 100 µL ALG solution) (n = 3).

### In vitro stimulation of BMDCs

BMDCs obtained from C57BL/6 mice were co-cultured with R837, NS (negative control) or LPS (positive control) as previously described [20]. Briefly, BMDCs were obtained from the femur and tibia of C57BL/6 mice, and cultured with 20 ng mL^−1^ rmGM-CSF (Xiamen Amoytop Biotech Co., Ltd., China) and 10 ng mL^−1^ rmIL-4 (Pepro Tech, USA). The medium was replaced three-quarters every 2–3 days until adherent cells were collected on the 8th day. The harvested immature DCs were resuspended in RPMI 1640 medium without cytokines and co-cultured with R837, NS or LPS for 48 h. Then incubated with 1 µL FITC-anti-mouse CD11c monoclonal antibody, PE-anti-mouse CD86 monoclonal antibody, and APC-anti-mouse CD80 monoclonal antibody (Biolegend, USA) for 30 min before evaluated by flow cytometry (Beckmann, BD Bioscience, USA).

### In vitro drug release from R837/Nab-PTX/ALG

R837/Nab-PTX/ALG was placed on the upper layer of a transwell plate at 37 ℃ and the lower layer was supplemented with normal saline. At different time points, normal saline was taken out to detect the concentration of Nab-PTX and R837 by ultraviolet spectrophotometer (310 nm and 270 nm respectively, Qingdao Jingcheng Instrument Co., Ltd, China), and new normal saline was added to keep the volume constant. The standard curves of Nab-PTX and R837 have been drawn via test by ultraviolet spectrophotometer in advance.

### CCK8 of Nab-PTX/ALG

100 µL cell suspension of human renal epithelial cells 293 T or mouse breast cancer cells 4T1 in 96 well plate was added 10 µL NS, Nab-PTX (10 mg mL^−1^), or Nab-PTX/ALG after pre-culture for 24 h (37 °C, 5% CO_2_). After incubation for 24, 48 and 72 h, each well was added 10 µL CCK8 solution respectively. When the solution turned light brown, we measured the absorbance at 450 nm using a microplate reader (Thermo Fisher Scientific, USA) for the first time until the maximum of OD_450_ reached 1 to 1.5.

### In vivo antitumor efficacy of R837/Nab-PTX/ALG

Two tumor models were established to evaluate the anti-tumor effect: subcutaneous murine hepatocellular carcinoma and breast cancer model. 2 × 10^6^ H22 cells in 100 µL PBS were subcutaneously injected into the lower right mammary gland of ICR mice to establish hepatocellular carcinoma model. 1 × 10^6^ 4T1 cells in 100 µL PBS were subcutaneously injected into the lower right mammary gland of Balb/c mice to establish breast cancer model. After 6 days later, when the tumor volume of the mice reached about 75 mm^3^, all mice were randomly divided into five groups: NS, ALG, R837/ALG, Nab-PTX/ALG, R837/Nab-PTX/ALG. The mice were administered with R837 (50 µg) and/or Nab-PTX (100 µg) on day 6 and day 13, which were dissolved in sodium ALG solution to a final volume of 100 µL per dose. The tumor size and body weight were measured every 2–3 days until the average volume of the NS group reached 1000 mm^3^, which was considered to have reached the standard for euthanasia of mice. 1 week after last treatment, main organs (heart, liver, spleen, lung and kidney) were excised for hematoxylin–eosin staining.

### Flow cytometry

One week after the last treatment, lymph nodes, spleens and tumors from murine hepatocellular carcinoma models were collected to detect the immune responses initiated by R837/Nab-PTX/ALG. Lymph nodes and spleens were ground, filtered to make single cell suspensions (0.5–1 × 10^6^ cells mL^−1^). Tumors were firstly cut into small pieces and incubated with collagenase type IV (1 mg/mL, Sigma, USA) at 37 ℃ for 3–4 h, before filtered to make single cell suspensions (0.5–1 × 10^6^ cells mL^−1^). All tested antigens are expressed on cell membranes, so all samples were stained with specific antibodies for 20 min in 4 °C in dark, and then washed before analysis. The following monoclonal antibodies (mAbs) were used for flow cytometry and purchased from Biolegend: CD11c-FITC (5 μg/mL), CD80-APC (2 μg/mL), CD86-PE (2 μg/mL), CD3-FITC (5 μg/mL), CD4-PerCP/Cy7 (2 μg/mL), CD8-PE-Cy5 (2 μg/mL), CD44-PE (2 μg/mL), CD62L-APC (2 μg/mL).

### Immunofluorescence staining

Tumors were obtained 1 week after last treatment from murine hepatocellular carcinoma models, made into paraffin sections, and incubated with calreticulin polyclonal antibody (1:100, Proteintech, USA) overnight at 4 °C. After washing, the sections were stained with goat anti-rabbit IgG H&L (Cy3, 1:200) (Abcam, UK) and DAPI (Sangon Biotech, China). After sealed with 50% glycerol, fluorescence pictures were taken by confocal microscope (Leica, German).

### RNA sequencing

Following the completion of the treatment regimen, tumors were excised 1week after the last treatment and quickly frozen by liquid nitrogen. The mRNA samples of the NS group and R837/Nab-PTX/ALG group were used for RNA sequencing (Berry Genomics, Beijing, China). Gene Ontology (GO) and Kyoto Encyclopedia of Genes and Genomes (KEGG) enrichment analysis used GOSeq (v1.34.1) and the database (http://en.wikipedia.org/wiki/KEGG) respectively.

### Statistical analysis

All statistical analysis was performed by GraphPad Prism 8.0.2 statistical software (San Diego, CA). All results are presented as mean ± SEM for at least three independent experiments. P values were analyzed by unpaired Student’s t-test, one-way ANOVA with Tukey’s multiple comparisons or Kaplan–Meier method as indicated. P < 0.05 was considered statistically significant.

## Results

### Preparation and characterization of the chemoimmunotherapy hydrogel

**Fig. 2:**
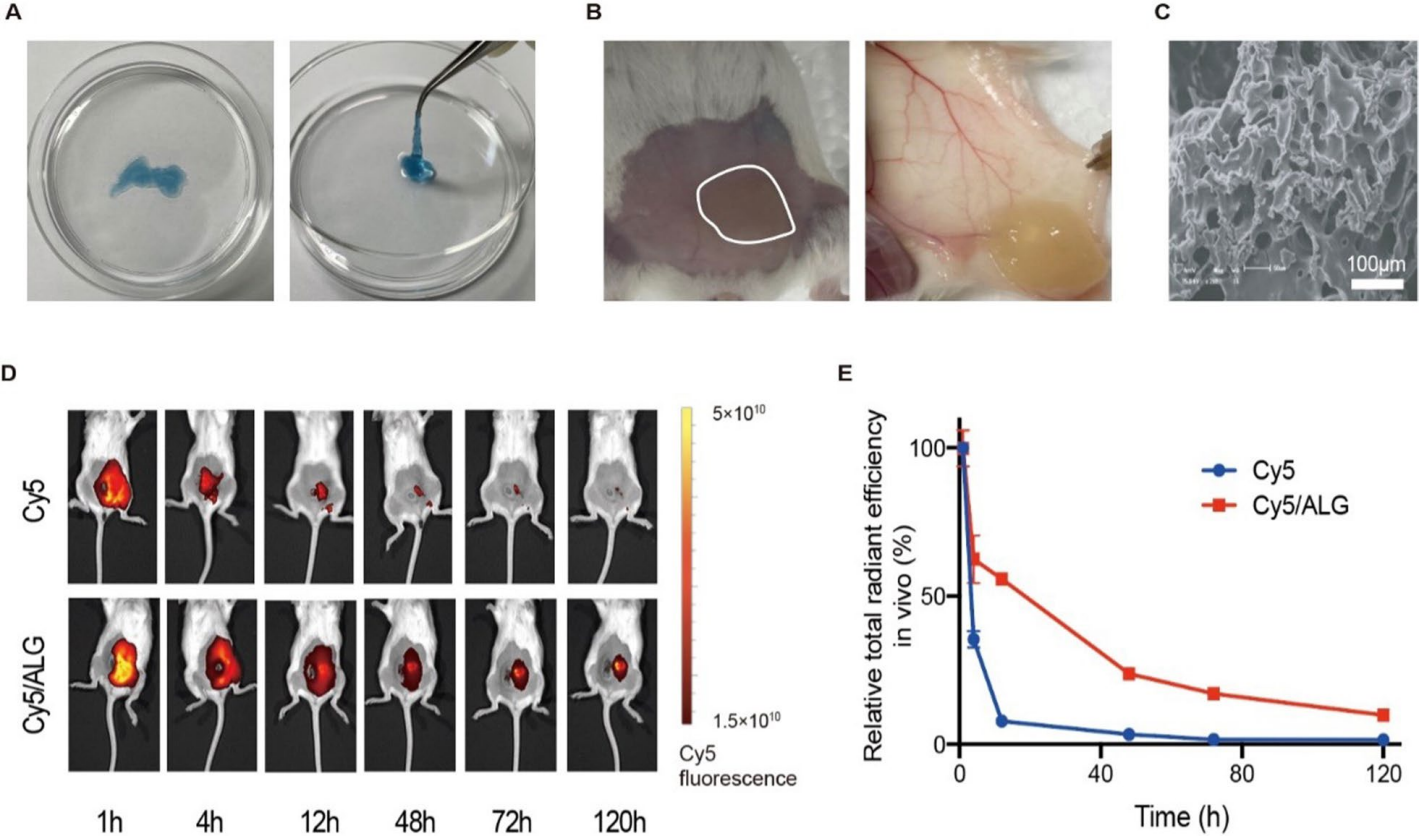
Preparation and characterization of the chemoimmunotherapy hydrogel. **A** Pictures of hydrogel containing sodium alginate (ALG) solution (20 mg mL^−1^), CaCl_2_ (1.8 × 10^–3^ mol L^−1^) and Cy5 (0.1 mg mL^−1^) formed in vitro at room temperature. **B** Pictures of hydrogels containing sodium ALG solution (20 mg mL^−1^) formed in vivo. **C** Scanning electron microscope images of blank ALG hydrogel (the scale bar is 100 µm). **D** Near-infrared (NIR) images of tumor-bearing ICR mice were taken on 1 h, 4 h, 12 h, 48 h, 72 h and 120 h, after peritumoral injection with pure Cy5 dye, or ALG hydrogel loaded with Cy5 (*n* = 3). **E** Relative total radiant efficiency of ICR mice after peritumoral injection with pure Cy5 dye, or ALG hydrogel loaded with Cy5 (*n* = 3). The error bars represented mean ± SEM

As previously reported, the injectable sodium ALG solution with the concentration of 20 mg mL^−1^ could rapidly form a hydrogel in the presence of multivalent cations in vivo [21, 22]. To observe the in vitro gelation, 1.8 × 10^–3^ mol L^–1^ CaCl_2_ (simulating the physiological concentration of Ca^2+^) was added to sodium ALG solution loaded with 0.1 mg mL^−1^ Cy5 dye, and the hydrogel was formed at room temperature within 10 s (Fig. 2A). In accordance with the literature, in vivo rapid gelation of 20 mg mL^−1^ sodium ALG solution after subcutaneous injection was shown in Fig. 2B. Scanning electron microscope images indicated that the loose structure of ALG hydrogel with a pore size of 20–50 µm was conducive to drug loading (Fig. 2C).

In our design, Nab-PTX and R837 are added into sodium ALG solution before gelation, then the drugs will be loaded in the pores of hydrogel scaffold as indicated in Fig. 1. Near-infrared (NIR) fluorescence imaging was used to detect the slow-release effect of hydrogel loaded with Cy5 dye, which simulated drugs such as Nab-PTX and R837 (Fig. 2D, E). The ALG hydrogel apparently prolonged the drug clearance time in situ after peritumoral injection in the murine hepatocellular carcinoma model. 55.77% fluorescence intensity remained 12 h post hydrogel injection, while only 7.83% remained after the injection of pure Cy5.

### In vitro function of the chemoimmunotherapy hydrogel

**Fig. 3:**
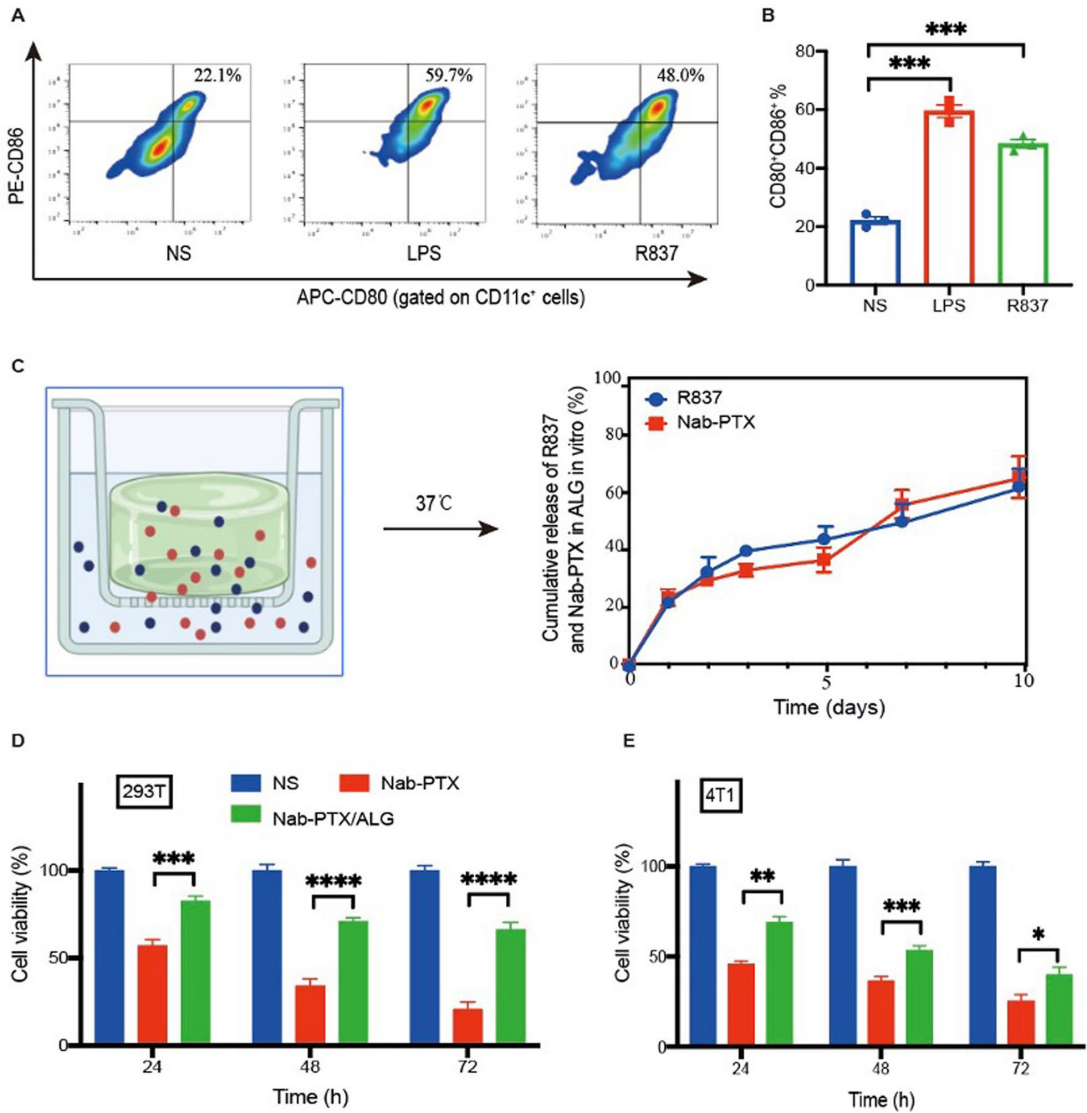
In vitro function of the chemoimmunotherapy hydrogel. **A** Representative flow cytometry images of mature DCs (mDCs, CD11c^+^CD80^+^CD86^+^) after co-incubation with R837 (5 µg mL^−1^), normal saline (NS, negative control), or LPS (1 µg mL^−1^, positive control) in vitro for 48 h. **B** The percentage of mDCs after co-incubation with R837, NS or LPS in vitro for 48 h (*n* = 3). The error bars represented mean ± SEM. *P*-values were calculated by two-tailed unpaired Student’s *t*-tests. ***P = 0.0004 (R837 vs NS) 0.0001 (LPS vs NS). **C** Cumulative release of R837 and nanoparticle albumin-bound-paclitaxel (Nab-PTX) from sodium alginate solution (ALG) based hydrogel in vitro at 37 ℃. **D** Cell viability of human renal epithelial cells 293 T after co-incubation with NS, Nab-PTX (10 mg mL^−1^), or ALG loaded with Nab-PTX (Nab-PTX/ALG) in vitro for 24 h, 48 h or 72 h respectively (*n* = 5). The error bars represented mean ± SEM. p-values were calculated by two-tailed unpaired Student’s *t*-tests. ***P = 0.0005, ****P < 0.0001. **E** Cell viability of mouse breast cancer cells 4T1 after co-incubation with NS, Nab-PTX (10 mg mL^−^.^1^), or ALG loaded with Nab-PTX (Nab-PTX/ALG) in vitro for 24 h, 48 h or 72 h respectively (*n* = 5). The error bars represented mean ± SEM. *p*-values were calculated by two-tailed unpaired Student’s *t*-tests. *P = 0.0312, **P = 0.004, ***P = 0.0017

Bone marrow-derived dendritic cells (BMDCs) of C57BL/6 mice were cultured and incubated with TL–R7 agonist R837. The proportion of mature DCs (CD11c^+^CD80^+^CD86^+^) was more than twofold upregulated by R837 compared with NS group (Fig. 3A, B). To simulate in vivo drug release from our hydrogel, we placed the chemoimmunotherapy hydrogel on the upper layer of a transwell plate at 37 ℃ and the lower layer was supplemented with normal saline (NS) (Fig. 3C). At different time points, NS was taken out to detect the concentration of Nab-PTX and R837 by ultraviolet spectrophotometer (310 nm and 270 nm respectively), and new NS was added to keep the volume constant. After 1 week of culture, only 49.84% R837 and 55.89% Nab-PTX were released from our hydrogel (Fig. 3C). Cell viability of human renal epithelial cells 293 T after incubation with Nab-PTX/ALG is significantly higher than incubation with Nab-PTX alone (Fig. 3D) while that of 4T1 murine breast cancer cells, one kind of so-called “cold” tumor, after incubation with Nab-PTX/ALG is similar to incubation with Nab-PTX alone (Fig. 3E). Hence, the slow-release effect of the chemoimmunotherapy hydrogel could reduce some damage to normal cells in vitro.

### In vivo antitumor effect of the chemoimmunotherapy hydrogel

**Fig. 4:**
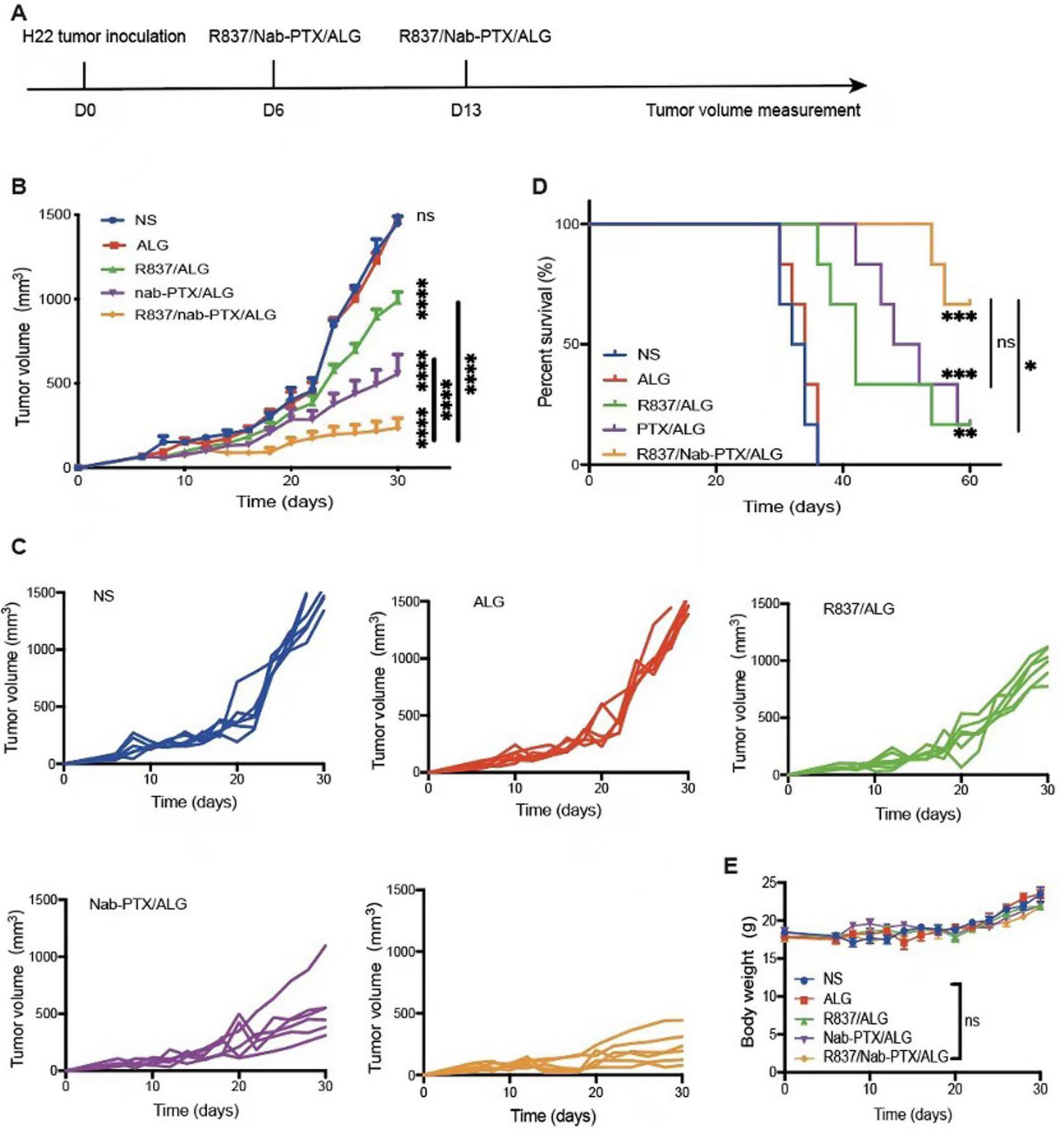
In vivo antitumor effect of the chemoimmunotherapy hydrogel. **A** Treatment schema of R837/Nab-PTX/ALG in tumor suppression experiment. 2 × 10^6^ murine hepatocellular carcinoma cells H22 in 100 µL PBS were injected subcutaneously on the lower right mammary gland of ICR mice to establish H22 tumor mouse models. **B** Average tumor-growth curves of ICR mice bearing H22 hepatocellular carcinoma with different treatments as indicated (*n* = 6). The mice were administered with R837 (50 µg) and/or Nab-PTX (100 µg) on day 6 and day 13, which were dissolved in normal saline or sodium ALG solution to a final volume of 100 µL per dose. The tumor size was measured every 2–3 days from the first day of administration until the average volume of the NS group reached 1000 mm^3^, which was considered to have reached the standard for euthanasia of mice. The error bars represented mean ± SEM and p-values were calculated by two-way ANOVA and Tukey post-test and correction. ns *P* > 0.05, ****P < 0.0001. **C** Tumor-growth curves of each mouse in different groups (*n* = 6). **D** Survival curves of ICR mice in different groups (*n* = 6). *P*-values were calculated by log-rank (Mantel–Cox) test, ns *P* > 0.05, *P = 0.0284, **P = 0.0013, ***P = 0.0006 (NS vs PTX/ALG, NS vs R837/Nab-PTX/ALG). **E** Average weight of different groups (*n* = 6). The error bars represent mean ± SEM and *p*-values were calculated by two-way ANOVA and Tukey post-test and correction, ns *P* > 0.05

To evaluate the antitumor efficacy of the chemoimmunotherapy hydrogel in vivo, especially in immune-infiltrated (“hot”) tumors, a subcutaneous H22 murine hepatocellular carcinoma model was established in female ICR mice (Fig. 4A). Six days after tumor inoculation by subcutaneous injection with 2 × 10^6^ H22 tumor cells into the lower right mammary gland of ICR mice, when the tumor volume reached about 75 mm^3^, R837 (50 µg) and/or Nab-PTX (100 µg) were dissolved in 100 µL normal saline or 20 mg mL^−1^ sodium ALG solution for peritumoral injection. Mice were randomly divided into five groups: NS, ALG, R837/ALG, Nab-PTX/ALG, R837/Nab-PTX/ALG. An additional treatment was administered 1 week after the first injection.

Obviously, the tumors of mice in the NS group and the ALG group grew quite rapid, while the tumor growth of other three groups (R837/ALG, Nab-PTX/ALG, R837/Nab-PTX/ALG) was significantly slower compared with NS group (Fig. 4B, C, p < 0.0001). Among all groups, R837/Nab-PTX/ALG treatment controlled the growth of tumors to the utmost extent. Figure 4C showed that in the R837/Nab-PTX/ALG group, all six mice had a slower growth trend of tumors, whose volumes were all below 500 mm^3^ about 1 month after tumor inoculation. Accordingly, the survival rate of R837/Nab-PTX/ALG group was 66.67% (4/6), which was much better than that of the R837/ALG group (1/6, P= 0.0284) and Nab-PTX/ALG group (1/6, *P* = 0.0515) on day-60 post tumor inoculation.

To further verify the antitumor efficacy, a subcutaneous 4T1 murine breast cancer model was established in female Balb/c mice (Fig. 7A). When the tumor volume reached about 75 mm^3^, Mice were randomly divided into five groups as before. Tumors in the R837/Nab-PTX/ALG group grew the slowest (Fig. 7B) and mice achieves the longest survival time (Fig. 7C).

### T cell responses and immunogenic cell death induced by the chemoimmunotherapy hydrogel

**Fig. 5:**
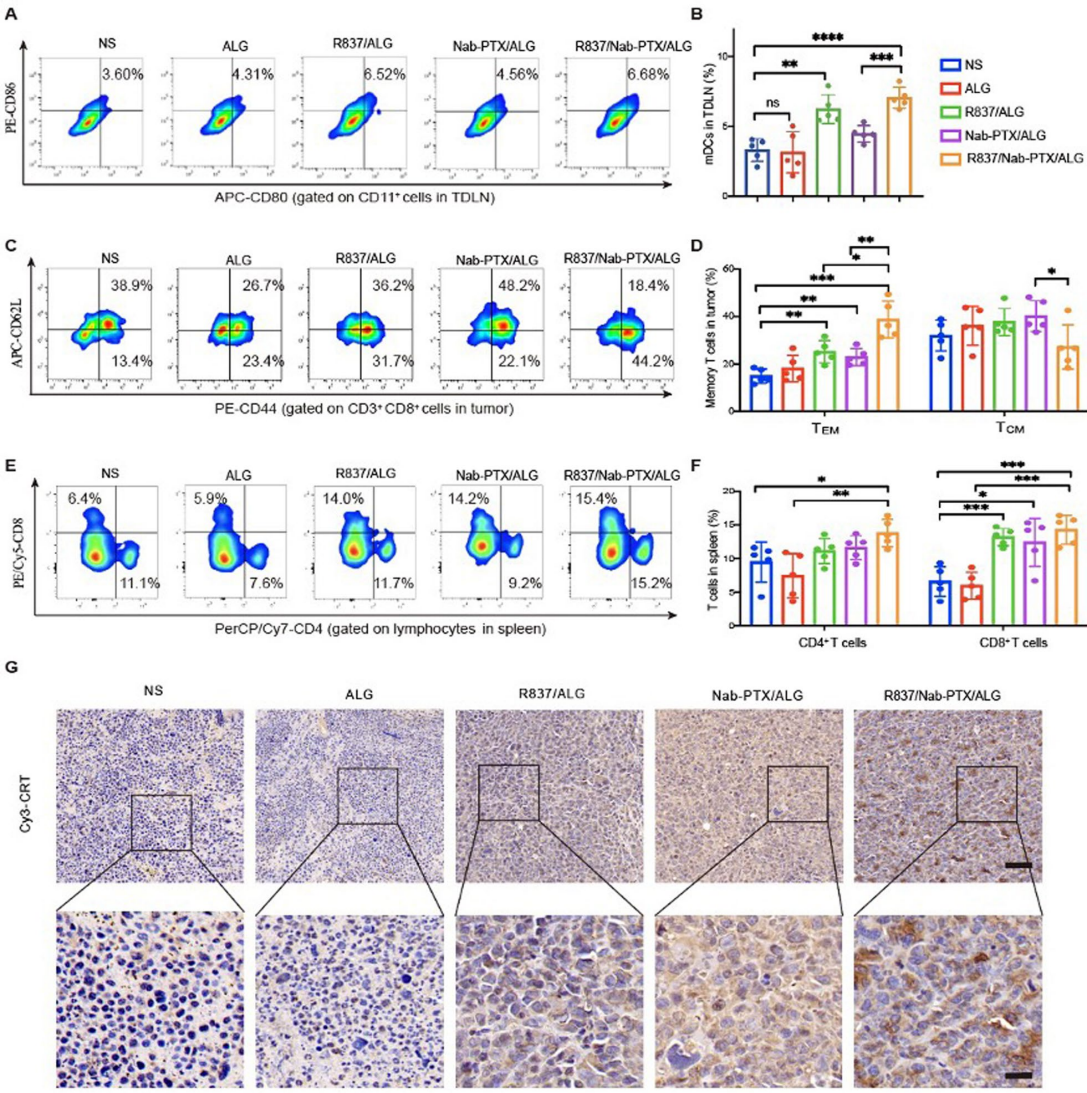
Immune responses and immunogenic cell death induced by the chemoimmunotherapy hydrogel. Representative flow cytometry images (**A**) and proportions (**B**) of mature DCs (mDCs, CD11c^+^CD80^+^CD86^+^) in tumor-draining lymph nodes (TDLNs) 1 week after the last treatment (*n* = 5). The error bars represented mean ± SEM. p-values were calculated by two-tailed unpaired Student’s *t*-tests. ns *P* > 0.05, **P = 0.0011 and ***P = 0.0003, ****P < 0.0001. Representative flow cytometry images (**C**) and proportions (**D**) of effector memory *T* cells (T_EM_, CD3^+^CD8^+^CD44^+^CD62L^–^) and central memory T cells (T_CM_, CD3^+^CD8^+^CD44^+^CD62L^+^) in tumors 1 week after the last treatment (*n* = 5). The error bars represented mean ± SEM. p-values were calculated by two-tailed unpaired Student’s *t*-tests. *P = 0.0102 (T_EM_) 0.0333 (T_CM_), **P = 0.0032 (NS vs R837/ALG) 0.0049 (NS vs Nab-PTX/ALG) 0.0033 (Nab-PTX/ALG vs R837/Nab-PTX/ALG) and ***P = 0.0002. Representative flow cytometry images (**E**) and proportions (**F**) of cytotoxic lymphocytes (CTLs, CD3^+^CD8^+^) and helper T cells (Th, CD3^+^CD4.^+^) in spleens 1week after the last treatment (*n* = 5). The error bars represented mean ± SEM. *p*-values were calculated by two-tailed unpaired Student’s *t*-tests. *P = 0.0276 (CD4) 0.0145 (CD8), **P = 0.0063 (CD4), ***P = 0.0004 (NS vs R837/ALG) 0.005 (ALG vs R837/Nab-PTX/ALG) 0.0002 (NS vs R837/Nab-PTX/ALG). (**G**) Calreticulin (CRT) staining of tumors (brown) 1 week after the last treatment (the scale bar is 62.5 µm and 20 µm, respectively)

In order to detect the immune responses induced by the chemoimmunotherapy hydrogel in vivo, we collected the inguinal lymph nodes, spleens, and tumors 1 week after last treatment, to acquire and analyze the immune cells by flow cytometry. In the inguinal lymph nodes, the proportion of mDCs (CD11c^+^CD80^+^CD86^+^) in these two groups (R837/ALG, R837/Nab-PTX/ALG) increased significantly as shown in Fig. 5A, B. Intratumoral T cells activated by mDCs showed a 2.30-fold increase of effector memory T cell (TEM, CD3^+^CD8^+^CD44^+^CD62L^−^) phenotype, which is capable of triggering strong anti-tumor effect by secreting TNF-α and interferon (IFN)-γ, rather than central memory T cell (T_CM_, CD3^+^CD8^+^CD44^+^CD62L^−^) phenotype (Fig. 5C, D) [23]. It was worth noting that the spleen cells of mice in the R837/Nab-PTX/ALG group exhibited highest proportions of cytotoxic lymphocytes (CTLs, CD3^+^CD8^+^) and helper T cells (Th, CD3^+^CD4^+^) in Fig. 5E, F. Both local and systemic T cell responses induced by R837/Nab-PTX/ALG were detected.

It is known that some chemotherapy drugs may induce ICD characterized by high expression of calreticulin (CRT) on the surface of dying cancer cells, thereby inducing effective immune responses. We studied the expression of CRT on the surface of cancer cells stained with anti-CRT antibody for confocal imaging. Obvious expression of CRT was observed in the Nab-PTX/ALG and R837/Nab-PTX/ALG group compared with other three groups, indicating that Nab-PTX would induce ICD of cancer cells (Fig. 5G).

**Fig. 6:**
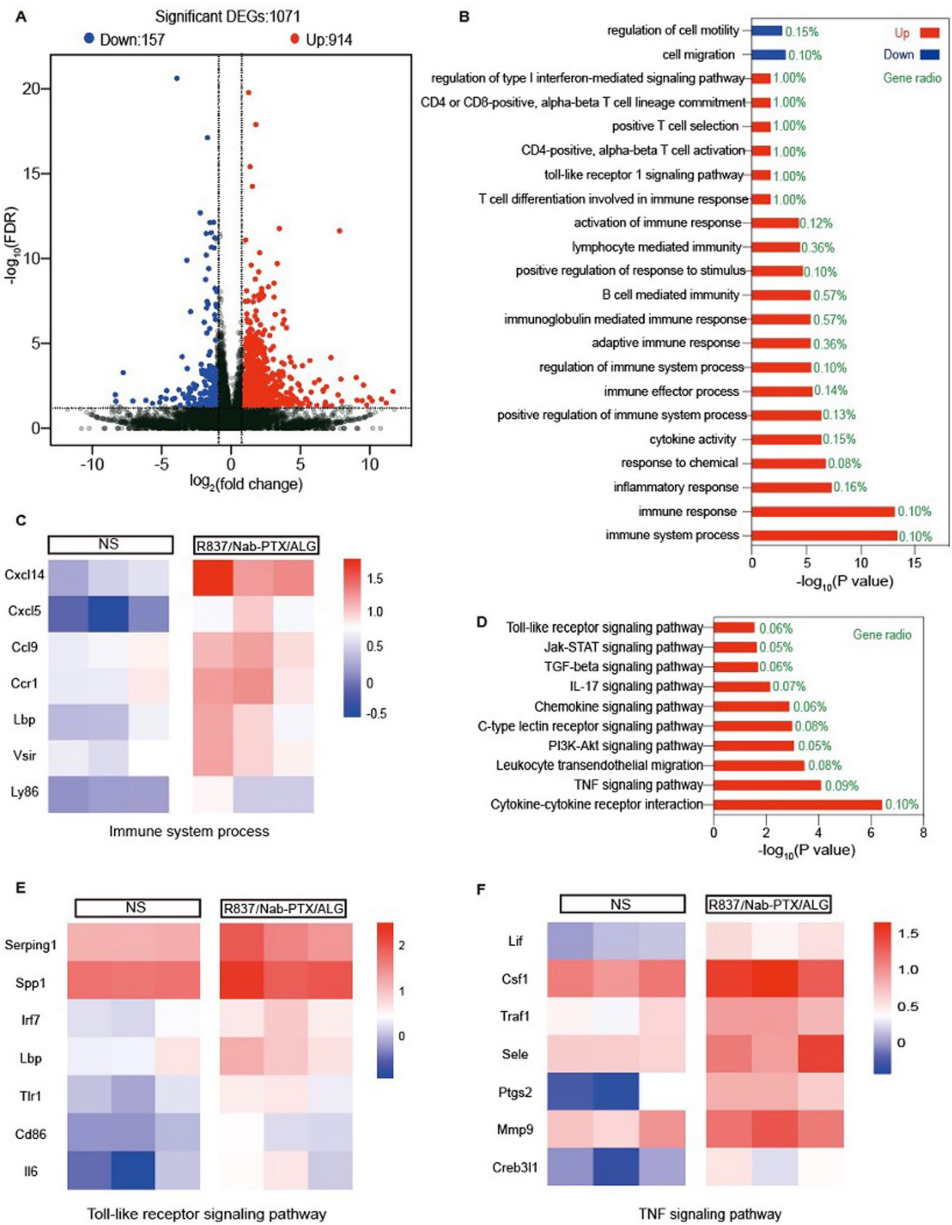
RNA-sequencing analysis. **A** Volcano map of significantly differentiated expression between NS and R837/Nab-PTX/ALG groups. The red dot represents significant up-regulated gene and the blue dot represents significant down-regulated gene. **B** Biological process and molecular function of GO enrichment analysis. **C** Differential gene expressions in immune system process by GO enrichment analysis. **D** Kyoto Encyclopedia of Genes and Genomes (KEGG) pathway analysis. **E**, **F** Differential gene expressions in Toll-like receptor signaling pathway and TNF signaling pathway

To further clarify the changes of tumor microenvironment after the chemoimmunotherapy hydrogel therapy, RNA sequencing (RNA-seq)-based transcriptome analyses was performed. There are 914 significantly up-regulated genes and 157 down-regulated genes between the R837/Nab-PTX/ALG group and NS group (Fig. 6A). GO enrichment analysis demonstrated that 20 biological processes related to immune responses upregulated by R837/Nab-PTX/ALG (Fig. 6B). Among them, the immune system process with the most significant difference, contains seven genes: cxcl14, cxcl5, ccl9, ccr1, lbp, vsir, ly86. KEGG enrichment analysis told more about signaling pathways (Fig. 5D). Many up-regulated genes were involved in numerous immune-related signaling pathways, such as TLR signaling pathway (Fig. 5E), TNF signaling pathway (Fig. 5F), Cytokine–cytokine receptor interaction, TGF-beta signaling pathway, PI3K-Akt signaling pathway and so on.

### Biosafety assessment

**Fig. 7:**
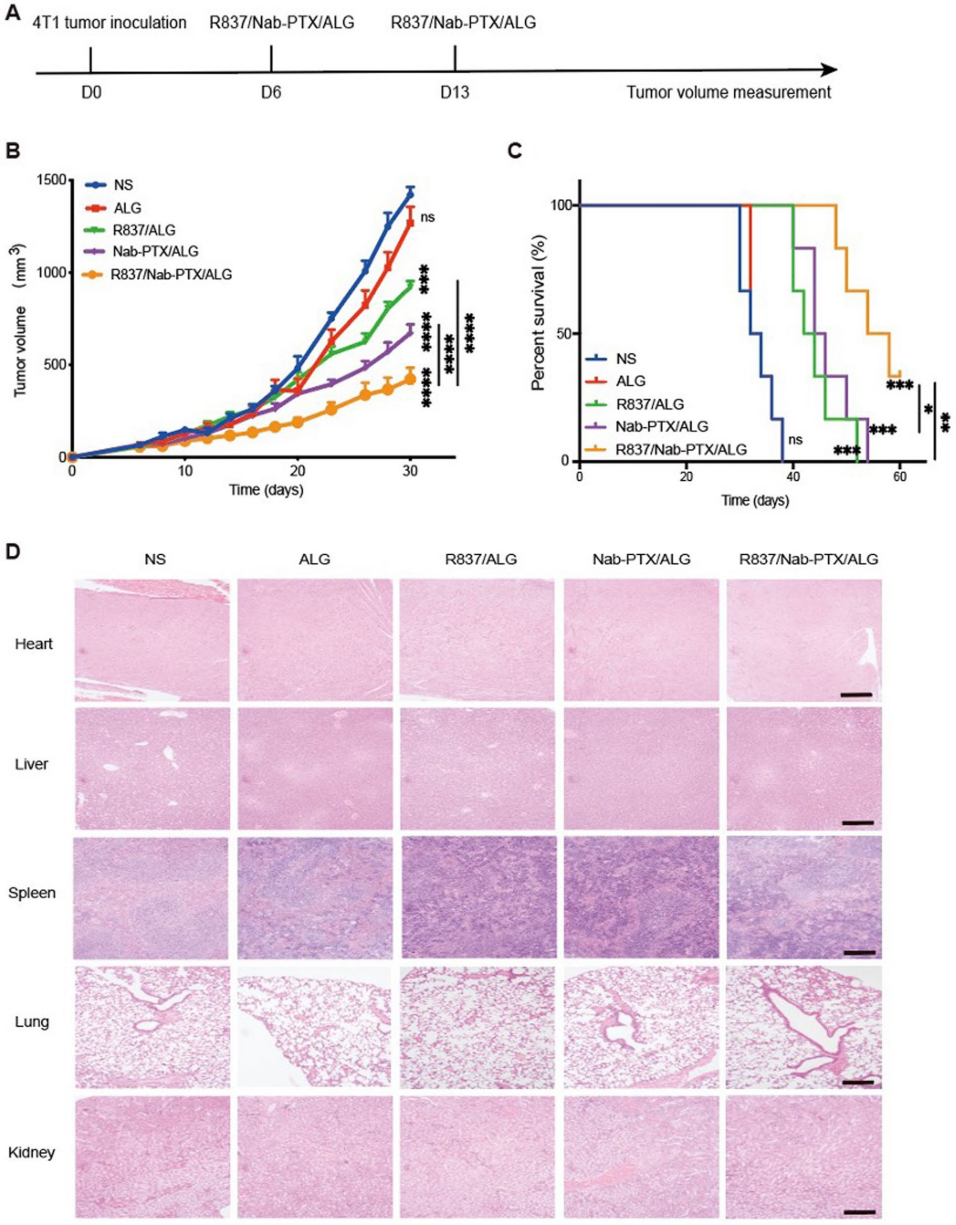
Antitumor effect and safety evaluation of the chemoimmunotherapy hydrogel. **A** Treatment schema of R837/Nab-PTX/ALG in tumor suppression experiment. 1 × 10^6^ murine breast cancer cells 4T1 in 100 µL PBS were injected subcutaneously on the lower right mammary gland of Balb/c mice to establish 4T1 tumor mouse models. **B** Average tumor-growth curves of Balb/c mice bearing 4T1 breast cancer with different treatments as indicated (*n* = 6). The mice were administered with R837 (50 µg) and/or Nab-PTX (100 µg) on day 6 and day 13, which were dissolved in normal saline or sodium ALG solution to a final volume of 100 µL per dose. The tumor size was measured every 2–3 days from the first day of administration until the average volume of the NS group reached 1000 mm^3^, which was considered to have reached the standard for euthanasia of mice. The error bars represented mean ± SEM and *p*-values were calculated by two-way ANOVA and Tukey post-test and correction. ***P = 0.0005, ****P < 0.0001. **C** Survival curves of Balb/c mice in different groups (*n* = 6). *P*-values were calculated by log-rank (Mantel–Cox) test, *P < 0.0194, **P < 0.0045, ***P = 0.0006 (NS vs R837/ALG, NS vs Nab-PTX/ALG, NS vs R837/Nab-PTX/ALG). **D** Hematoxylin–eosin staining of main organs, including heart, liver, spleen, lung and kidney. (the scale bar is 200 µm)

When the R837/Nab-PTX/ALG was applied to the treatment of H22 murine hepatocellular carcinoma model, compared with the NS group, the mice in the other four groups did not experience abnormal weight fluctuations (Fig. 4E). Hematoxylin–eosin staining of main organs taken out 1 week after the last treatment were shown in Fig. 7D and there was no obvious damage found in all groups. Therefore, the injectable chemoimmunotherapy hydrogel was examined with good biological safety.

## Discussion

Here, we provide a novel chemoimmunotherapy based on a chemotherapeutic drug Nab-PTX and an immunostimulating agent R837, which showed great tumor inhibition effect in different tumor models. The unique advantage of our chemoimmunotherapy strategy is to make the tumor a “in situ vaccine” after peritumoral administration of the ALG hydrogel loaded with Nab-PTX and R837. The rapid gelation of injected solution is crucial (Fig. 2A, B), which allowed sustained release and tumor retention of Nab-PTX and R837 in vivo (Figs. 2D, E, 3C). Not only ICD inducer Nab-PTX, but also the immune adjuvant R837, is critical in our design, and could greatly enhance the tumor-specific immune responses.

In accordance with the literature mentioned above, we found Nab-PTX could also induce ICD similar with normal PTX (Fig. 5G). Nab-PTX was the first nano-formulation of PTX to be developed and has been approved for the treatment of breast cancer, non-small cell lung cancer, and pancreatic adenocarcinoma [24]. Compared to normal formulation, Nab-PTX with good colloidal stability in vivo, reduces the systemic side effects related to hypersensitivity reactions and delivers more PTX to the tumor site [25]. To make immunogenic antigens released by Nab-PTX efficiently activate DCs and further induce tumor specific T cells, an immunostimulating agent is necessary. PTX is also reported to capable of binding to TLR4 and activating downstream signaling pathways for secretion of proinflammatory cytokines and upregulation of immune cells [26]. But from its moderate clinical efficacy of single drug, the antitumor immune response initiated by PTX itself is not enough. Hence, TLR7 agonist R837, the first TLR agonist that approved for cancer treatment, is picked out by us to strengthen the co-stimulating signal [15].

Both flow cytometry and RNA-seq were used to analyze the tumor microenvironment after R837/Nab-PTX/ALG treatment. Compared with NS group, significant upregulation of cd86 gene (symbol of mature DC) was detected due to activation by immunogenic antigens released by tumor cells after apoptosis induced by Nab-PTX, and by TLR signaling pathway sensitized by R837 (Fig. 6E). Tumor ICD exposed damage-associated molecular patterns CRT (Fig. 5G), which have been considered to be closely relevant to facilitate DC maturation and antigen presentation to naive T cells [27]. Then mature DCs migrate to lymph nodes to induce T_EM_, since we found the proportions mature DCs upregulated in the tumor draining lymph nodes (Fig. 5A, B). Both increased CTLs and Th cells were detected in spleens as well (Fig. 5C, D). Finally compared with NS group, 2.30-fold increase of intratumoral T_EM_ properties in the R837/Nab-PTX/ALG group (Fig. 5E, F), indicated that activated T cells eventually infiltrated to the tumor microenvironment to kill tumor cells. A considerable number of chemokines and related receptors involved in the above immune system process were also significantly upregulated (Fig. 6C).

However, we also found the activation of TNF signaling pathway in the tumor microenvironment. TNF has been demonstrated to have the double-edged sword effect on tumor growth [28, 29]. On one hand, it can inhibit tumor cell growth by inducing tumor cell apoptosis. But on the other hand, it also stimulates the proliferation, survival, migration and angiogenesis of some cancer cells that are resistant to TNF-induced cytotoxicity, leading to the development of tumor. More and more evidence shows that, in the process of cancer progression, TNF can not only reduce the effect of immunotherapy by directly regulating the activation, function and survival of T cells, but also change the phenotype of cancer cells, make it express immunosuppressive molecules and invisible to T cells, contributing to immune escape [30, 31]. Deeply analyzing the specific function of TNF signaling pathway in tumor microenvironment may help us to further improve our chemoimmunotherapy hydrogel treatment.

Both peritumoral administration and ALG hydrogel require relatively lower doses of drugs and less frequency of injection, minimizing the toxicity on normal cells and normal tissues. In multiple mouse models, such as murine hepatocellular carcinoma and breast cancer model, R837/Nab-PTX/ALG treatment showed the greatest control of the tumor growth compared with other groups and more than half of mice survived about two months after tumor inoculation, which may indicate the form of immune memory to inhibit the tumor growth for a long time. It is worthy of further investigation about the memory subsets of T cells. Furthermore, the synthetic materials of this hydrogel have good biocompatibility, and the preparation process is feasible and safe. All above give this strategy great potential of clinical translation.

## Conclusions

In this study, we successfully constructed an injectable chemoimmunotherapy hydrogel based on a chemotherapeutic drug Nab-PTX and an immunostimulating agent R837. The hydrogel formed near the tumor and slowly released Nab-PTX and R837 into the tumor site to make it a “in situ vaccine”. The tumor itself exposed immunogenic antigens due to ICD induced by Nab-PTX and greatly promote the DCs maturation with the help of R837. Notably, the hydrogel increased the number of T_EM_ by 23–folds, greatly enhancing the anti-tumor efficacy. We verified its antitumor efficacy in the subcutaneous mouse ectopic model of both murine hepatocellular carcinoma and breast cancer model. These findings provide a feasible strategy for localized chemoimmunotherapy.

## Data Availability

The data that support the findings of this study are available from the corresponding author, upon reasonable request.

## Abbreviations

Nab-PTX: Nanoparticle albumin-bound-paclitaxel
ICD: Immunogenic cell death
CRT: Calreticulin
DAMP: Damage-related molecular pattern
HMGB1: High-mobility group box 1
TLR7: Toll-like receptor 7
CpG-ODNs: CpG oligodeoxynucleotides
ALG: Alginate
T_EM_: Effector memory T cells
GO: Gene Ontology
KEGG: Kyoto Encyclopedia of Genes and Genomes
BMDCs: Bone marrow derived dendritic cells
NS: Normal saline
